# Effects of Huang-Lian-Jie-Du Decoction on Oxidative Stress and AMPK-SIRT1 Pathway in Alzheimer's Disease Rat

**DOI:** 10.1155/2020/6212907

**Published:** 2020-01-02

**Authors:** Xinru Gu, Haiyu Zhao, Junyi Zhou, Yanyan Zhou, Xiaolu Wei, Hongjie Wang, Baolin Bian, Jian Yang, Wei Ren, Nan Si

**Affiliations:** ^1^Institute of Chinese Materia Medica, China Academy of Chinese Medical Sciences, Beijing 100700, China; ^2^Drug Research Center of Integrated Traditional Chinese and Western Medicine, Affiliated Traditional Chinese Medicine Hospital, Southwest Medical University, Luzhou 646000, China

## Abstract

Huang-Lian-Jie-Du Decoction (HLJDD), traditional Chinese medicine (TCM), is proven to have ameliorative effects on learning and memory deficits of Alzheimer's disease (AD). The current study aims to reveal the underlying mechanism of HLJDD in the treatment of AD by simultaneous determination on the regulation of HLJDD on oxidative stress, neurotransmitters, and AMPK-SIRT1 pathway in AD. AD model rat was successfully established by injection of D-galactose and A*β*_25-35_-ibotenic acid. Morris Water Maze (MWM) test was used to evaluate the success of AD modelling. On this basis, an advanced technique with UPLC-QqQ MS/MS was built up and applied to determine the levels of 8 neurotransmitters in rat plasma. Significant alternation in methionine, glutamine, and tryptophan was observed in AD rats' plasma after the administration of HLJDD, relative to the model group. Meanwhile, HLJDD could upregulate the levels of SOD, GSH-Px, AMPK, and SIRT1 and downregulate the content of MDA in the peripheral system of the AD rats. The underlying therapeutic mechanism of HLJDD for the treatment of AD was associated with alleviating oxidation stress, inflammation, neurotransmitters, and energy metabolism. These data provide solid foundation for the potential use of HLJDD to treat AD.

## 1. Introduction

HLJDD, a classical TCM formula used for heat clearance and detoxification, consists of *Rhizoma coptidis* (Rc), *Radix scutellariae* (Rs), *Cortex phellodendr* (Cp), and *Fructus Gardeniae* (Fg) with a weight ratio of 3 : 2 : 2 : 3. In clinic, HLJDD has been used to treat AD in China and other Asian countries recently [1, 2]. Our previous study showed that alkaloids, flavonoids, and iridoids were the main bioactive ingredients of HLJDD that exhibited different mechanisms of action in the anti-inflammatory process [3, 4]. Recently, HLJDD has made remarkable achievement in the treatment of AD with the specific characteristics of anti-inflammation, oxidation resistance, preserving energy metabolism, reducing the production of amyloid beta-peptide (A*β*), and improving memory in AD mice [5–8]. However, much attention has been paid to the influence of HLJDD on the central nervous system while few research focuses on the regulation of the peripheral system of AD by HLJDD.

AD is an age-related neurodegenerative disorder involving behavioural changes and difficulty in thinking [9, 10]. The major pathological hallmarks of AD are neuronal loss, amyloid senile plaques, and neurofibrillary tangles (NFTs) within the cerebral cortex. Guedes et al. reported that the accumulation of diverse forms of A*β* in the AD brain occurred before the development of visible senile plaques and formation of senile plaques and NFTs [11]. The presence of methionine at 35 position of Aβ is critical to Aβ-induced oxidative stress and neurotoxicity [12–14]. The activities of antioxidant enzymes including glutathione peroxidase (GSH-Px) and catalase displayed an age-dependent decline in both brain and plasma [15]. Neurons, which contribute to 50–80% of overall energy balance of the whole brain with glucose oxidative metabolism, are thought as a principal energy source and are easily vulnerable to diverse pathologic inputs that limit the supplement of the energy [16–18]. Neurotransmitters play an important role in the various activities of central and peripheral nervous systems and show different contents between normal people and patients with AD [19]. The interaction of A*β* with mononuclear phagocytes, including microglia and recruited peripheral blood monocytes, would further induce neuroinflammation. Consequently, the occurrence of oxidative stress, disturbance of the energy metabolism, and varied levels of the neurotransmitters are closely related to the development of AD.

AMP-activated protein kinase (AMPK), a key kinase involved in regulating cell energy metabolism, is an important regulator of Aβ generation [20, 21]. Short-term exposure of cultured rat hippocampal neurons to A*β* oligomers transiently decreases intracellular ATP levels and AMPK activity [22]. When confronted with inflammation, the combination of AMPK with silent information regulator of transcription 1 (SIRT1) could exert synergistic effects to jointly maintain the energy homeostasis [22, 23]. Accumulative findings indicate that increasing in oxidative stress during aging could decrease the catabolic activity of SIRT1, possibly by reactive oxygen [24]. Therefore, AMPK-SIRT1 may be potentially involved in the pathogenesis of AD.

HLJDD, as well as its major components, has ameliorative effects on learning and memory deficits. However, the therapeutic mechanism is still unclear. In this study, the rats injected with D-galactose and A*β*_25-35_-ibotenic acid were selected as AD model and this aims to explain the potential mechanism of HLJDD in treating AD rats from a new perspective. To the best of our knowledge, this is the first report on the investigation of the therapeutic mechanism of HLJDD on AD rats from the perspective of peripheral oxidative stress, inflammation, energy metabolism, and neurotransmitters.

## 2. Material and Methods

### 2.1. Chemicals and Regents

Rs, Rc, Cp, and Fg were obtained from their geoauthentic product areas. These four herbal medicines were authenticated as *Scutellaria baicalensis* Georgi (voucher specimen number: SB-0315), *Coptis chinensis* Franch (voucher specimen number: CC-0311), *Phellodendron chinense* Schneid (voucher specimen number: PC-0311), and *Gardenia jasminoides* Ellis (voucher specimen number: GJ-0311), respectively, by professor He Xi-Rong (Institute of Chinese Materia Medica, China Academy of Chinese Medical Sciences) [3, 25–27]. The reference standards diazepam, serotonin, glutamate, creatinine, arginine, A*β*_25-35_, and ibotenic acid were purchased from Sigma (St, Louis, MO, USA). Among them, diazepam was used as the internal standards (S1). Tryptophan and methionine were obtained from Accelerating Scientific and Industrial Development thereby Serving Humanity (Beijing, China). Adrenaline and glutamine were provided by The National Institute for the Control of Pharmaceutical and Biological Products (Beijing, China). HPLC grade methanol and acetonitrile for the qualitative analysis and extraction were obtained from Honeywell Burdick and Jackson (Swedesboro, NJ, USA). HPLC grade formic acid was provided by Thermo Fisher Scientific (Bremen, Germany), and ultrapure water was purified by Millipore system (Millipore, Billerica, MA, USA). Other chemicals and solvents were of analytical grade.

### 2.2. Formula Preparation

In the preparation of HLJDD, four samples of dried plants were grinded into powders and mixed in a ratio of 2 : 3 : 2 : 3 (Rs : Rc : Cp : Fg), further decocted twice with boiling water (1 : 10, w/v) for 2 h. Then, the aqueous extract was concentrated and dried on a rotary vacuum evaporator at 80°C.

### 2.3. Surgical Procedure

Wistar rats (male, 270 ± 20 g), purchased from Cisco North Biotechnology Co, Ltd. (Beijing, China), were grown up in an environmentally controlled room (12 h light cycle) at 20 ± 1°C and 50 ± 10% relative humidity and feed with constant access to rodent chow (Nanjing, China) and water. A certain number of rats were randomly selected for subcutaneous injection of 50 mg/kg D-galactose for 45 days, which was dissolved in 0.9% saline (20 mg in 2 mL). The control group (*n* = 6) received subcutaneous injection of the same volume of saline. In the forty-sixth day, rats were randomly divided into a sham group (*n* = 5) and AD's model group (*n* = 20). A*β*_23-35_ was dissolved in 0.9% saline and incubated for 7 days at 37°C; then ibotenic acid was added to form the A*β*_25-35_-ibotenic acid solution (4.0 mg/mL A*β*_23-35_ and 2.0 mg/mL ibotenic acid). Sodium pentobarbital (4%, 40 mg/kg) was intraperitoneally injected before surgery. A*β*_25-35_-ibotenic acid solution (2 *μ*L) was administered into the nucleus basalis magnocellularis (NBM) over a period of 5 min, and then the needle was left in place for 10 min after the infusion. Rats in the sham group were injected with 2 *μ*L of 0.9% saline by the same procedure. After the operation, all rats received subcutaneous injection of benzylpenicillin sodium immediately to prevent infections. The ethics committees of Cisco North Biotechnology Co., Ltd. (Beijing, China) and the China Academy of Chinese Medical Sciences (Beijing, China) approved the experimental protocol. The ethical approval number was BJAM2016052105.

### 2.4. Morris Water Maze (MWM) Test

Seven days after the surgical procedure, all rats underwent a spatial learning and memory test using the MWM test [15, 26] with tiny modification, as used for screening. In navigation experiment which contains four test sessions per day for continuous four days, the interval between each test sessions was 45 minutes. For each assay, rats were allowed to swim until they found and landed on the platform for 15 sec. If they failed within 90 sec, the rats were picked up and placed on the platform for 15 sec. At the end of the last training, the platform was removed and the rats were placed from a fixed location. Then the swimming distance and time were recorded using video tracking.

### 2.5. Animal Grouping and Sample Collection

After the success of AD modelling, the remaining AD rats (*n* = 12, the postoperative survival rate was 60%) were divided into two groups at random: AD rats with HLJDD for one week (HLJDD one week group, *n* = 6) and AD rats with physiological saline group (model group, *n* = 6). The other two groups were sham group (*n* = 4) and control group (*n* = 6). The prepared HLJDD extract power (HLJDD-EP) solution was administered to the rats by oral gavage for one week at 2 mL/100 g body weight (crude material content: 3.5 g/kg/d). The sham group, model group, and control group received the same dose of physiological saline by oral gavage.

### 2.6. Determination of Superoxide Dismutase (SOD), Malondialdehyde (MDA), and GSH-Px in Rat's Hepar, Spleen, Kidney, and Plasma

The excised hepar, spleen, and kidney tissues were homogenized with ice-cold saline and centrifuged at 2500 rpm for 10 min to obtain the supernatant. The expressions of SOD, GSH-Px, and MDA in plasma, hepar, spleen, and kidney tissues were measured by spectrophotometer kits (SOD, GSH-PX, and MDA checkerboard were purchased from Nanjing Jiancheng Biotechnology Co., Ltd (Nanjing, China)). All of the procedures were performed by the same operator according to the manufacturer's protocol.

### 2.7. Determination of 8 Neurotransmitters in Rat's Plasma by UPLC-QqQ MS/MS

#### 2.7.1. Preparation of Standards Solutions and Plasma Samples

Eight reference standards including serotonin, glutamate, creatinine, arginine, tryptophan, methionine, adrenaline, and glutamine were dissolved in 50% methanol and diluted with 50% methanol (containing 0.1% formic acid) to a series of concentrations. An internal standard stock solution was also prepared with 50% methanol. Aliquots of 100 *μ*L from plasma were mixed with 10 *μ*L of ascorbic acid (dissolved in physiological saline, w/v: 1 g/100 mL), 10 *μ*L of IS, and 380 *μ*L of methanol (containing 0.2% formic acid), respectively. Followed by vortex and centrifugation at 12000 rpm for 15 min, the aliquots were analyzed by UPLC-QqQ MS/MS.

#### 2.7.2. Chromatographic Conditions

An Agilent 6490 triple quadrupole LC-MS system (Agilent Corporation, MA, USA) equipped with G1311 A quaternary pump, G1322 A vacuum degasser, G1329 A autosampler, and G1316 A thermostat was used for the UPLC-QqQ MS/MS analysis. The mobile phase consisted of acetonitrile containing 0.05% formic acid (solvent A) and water containing 20 mmol ammonium acetate (solvent B). The stepwise linear gradient was optimized as follows: 0–20 min, linear from 95% to 70% A; 20–21 min, linear from 70% to 50% A; 21–24 min, held at 50% A; 24–25 min, linear from 50% to 95% A; and 25–30 min, held at 95% A for equilibration of the column. The flow rate was 0.3 mL/min. The injection volume was 3 *μ*L. The separation was achieved at 25°C using an optimized Waters ACQUITY UPLC BEH Amide column (2.1 mm × 100 mm, 1.7 *μ*m).

**Figure 1:**
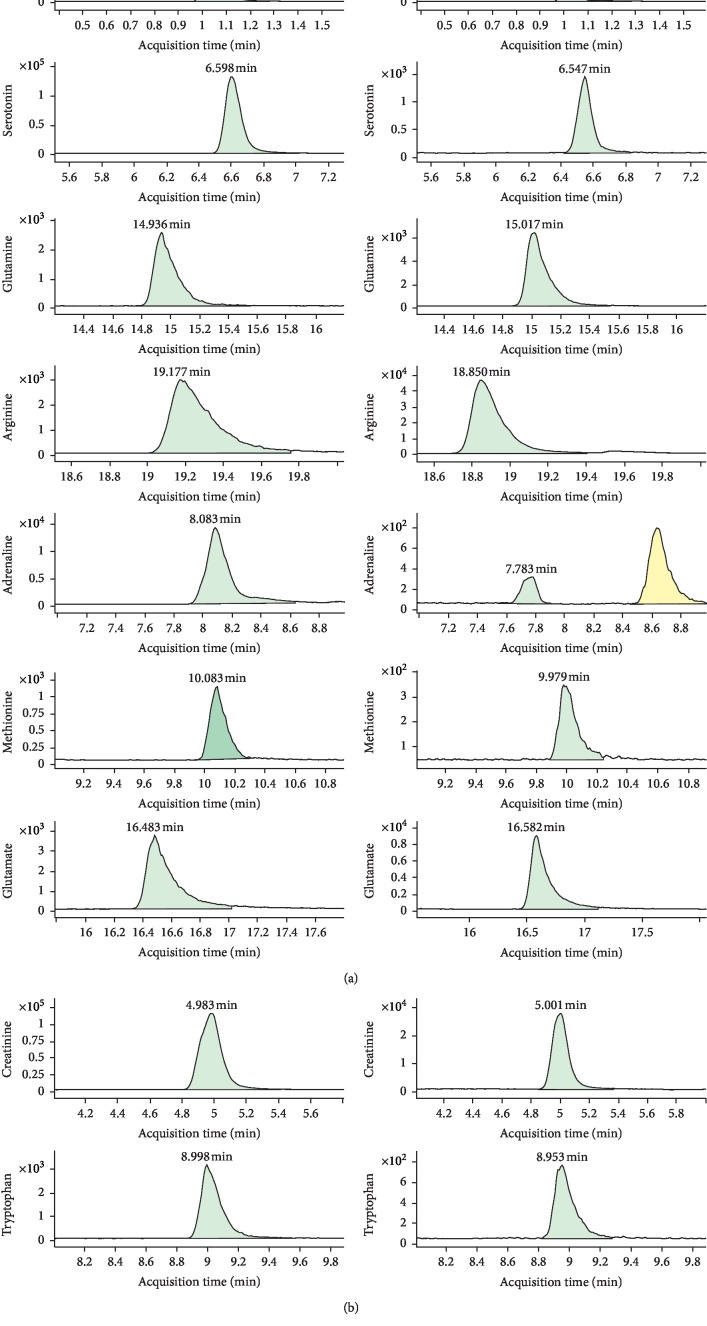
The extracted chromatograms of 8 constituents and internal standards in mixed reference substance and plasma.

The analytes were determined by monitoring the precursor-product transition in the MRM mode using ion polarity switching mode. To ensure the desired abundance of each compound, the CE values and other parameters were optimized and were as follows: cycle time, 300 ms; gas temp, 200°C; gas flow, 14 L/min; nebulizer, 20 psi; sheath gas flow, 11 L/min; capillary voltage, 3 kV; nozzle voltage, 1.5 kV; and Delta EMV(+), 200 V. The optimized mass transition ion pairs (*m/z*) and CE values for neurotransmitters are shown in Table 1. The MRM chromatograms of 8 neurotransmitters are shown in Figure 1.

### 2.8. Detection of AMPK and SIRT1 in Rat's Hepar, Spleen, and Kidney with Western Blot and Real-Time-PCR

#### 2.8.1. Western Blot

Excised hepar, spleen, and kidney tissues were homogenized in ice-cold saline with a SCIENTZ glass homogenizer (DY89-1). Further, 200 *μ*L of cell lysis buffer was added to the slurry containing 10 mg of tissue and incubated on ice for 15 min. Then, the supernatant was separated by centrifugation (12000 rpm for 10 min at 4°C) and stored at −20°C. Protein concentrations were determined by BCA assay kit. After SDS-PVDF, proteins were transferred from gel to nitrocellulose membranes. Membranes were blocked in 5% no fat dried milk in TBST (1.65 mL of 20％ Tween was added to 700 mL TBS) for 1 h and then incubated overnight with the specific antibody of the AMPK (Ab32047, Abcam, UK) and SIRT1 (Ab12193, Abcam, UK). After incubation with the relative second antibody, immunoreactive bands were quantified using imaging system (EUV-LDUV, Korea Biotech). Values were corrected with the absorbency of the *β*-actin (4957 CST, Cell Signaling, China).

#### 2.8.2. Real-Time PCR

Total RNA was extracted from hepar, spleen, and kidney using standard Trizol RNA isolation method. Reverse transcription of 10 *μ*g RNA was carried out. The qualities of RNA and cDNA were checked using 2720 nucleic acid analyzer (ABI, USA). Special primers designed against rat SIRT1 and AMPK subunit were verified in NCBI Blast. Primers against rat *β*-Actin were used as the internal control. Sequences of the primers along with their annealing temperature are shown in Table 2. The total reaction volume was 10 *μ*L, and 1 *μ*L cDNA was used as the template. Fluorescence was detected using Roche Light Cycler® 480II Detection System. PCR products were visualized with gel electrophoresis to confirm a single product of the correct size. Ratios of the target gene to *β*-actin were calculated and compared between samples.

### 2.9. Statistical Analysis

All values measured were presented as means ± SD. Statistical significance was determined by one-way ANOVA followed by Fisher's LSD test or Student's *t*-tests. A *p* value less than 0.05 was considered statistically significant.

## 3. Results

### 3.1. Morris Water Maze (MWM) Test

**Figure 2:**
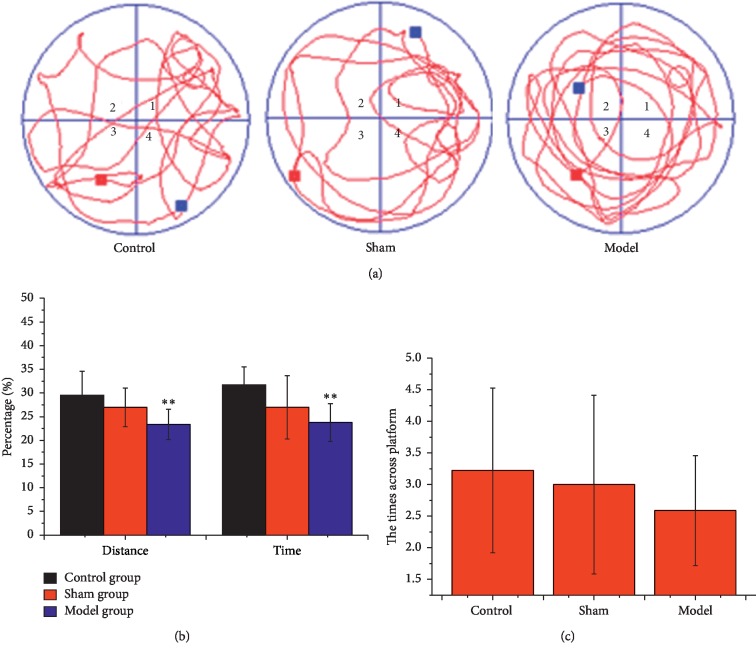
Effects of D-galactose and A*β*_25-35_-ibotenic acid injection on memory loss in rats. (a) The real-time monitoring of rats motion track Morris Water Maze test experiment. (b) Changes in the distance percentage and the time percentage and (c) the times across platform in the target quadrant. All the results are expressed as mean ± SD. *n* = 10; ^*∗*^*p* < 0.05, ^*∗∗*^*p* < 0.01 (comparison with control group).

The swimming distance and time were recorded in the MWM test using video tracking (Figure 2). Data was analyzed by MWM software. The times across the platform showed a decreased tend in both the sham group and the model group. Compared with the control group, the times across the platform, the distance percentage, and time percentage of the sham group decreased slightly while decreased significantly in the model group (*p* < 0.01), demonstrating successful establishment of AD model rats.

### 3.2. Variation of SOD, GSH-PX, and MDA between Different Groups

**Figure 3:**
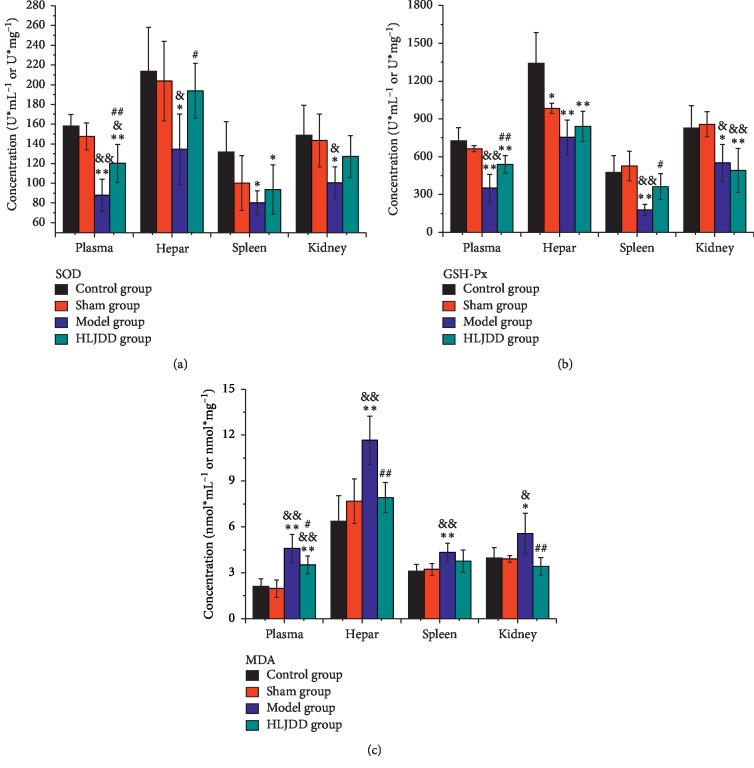
Effects of HLJDD on oxidative stress index in peripheral tissue of AD rats (*n* = 5). (a) The concentration of SOD, (b) GSH-PX, and (c) MDA in rats' plasma, hepar, spleen, and kidney. ^*∗*^*p* < 0.05, ^*∗∗*^*p* < 0.01 (comparison with control group); ^&^*p* < 0.05, ^&&^*p* < 0.01 (comparison with sham group); ^#^*p* < 0.05, ^##^*p* < 0.01 (comparison with model group).

The oxidative stress related substances including SOD, MDA, and GSH-Px were measured in the present study. Compared with the control group, the contents of SOD in the plasma, hepar, spleen, and kidney displayed the same downward trend in the sham group (Figure 3(a)), especially in the model group (plasma: *p* < 0.01; hepar, spleen, and kidney: *p* < 0.05). After the gastric gavage of HLJDD for one week, the level of SOD was improved in different degrees (plasma: *p* < 0.01; hepar: *p* < 0.05). The concentration of GSH-Px in diverse tissues showed different variations in the sham group (Figure 3(b)) but displayed dominant difference in the model group (plasma, hepar, and spleen: *p* < 0.01; kidney: *p* < 0.05). After treating with HLJDD, the contents of GSH-Px went up except in kidney (plasma: *p* < 0.01; spleen: *p* < 0.05). MDA, an important product of lipid peroxidation, presented a slight change in the sham group (Figure 3(c)) but increased significantly in the model group (plasma, hepar, and spleen: *p* < 0.01; kidney: *p* < 0.05) and turned back after the administration of HLJDD (plasma: *p* < 0.05; hepar and kidney: *p* < 0.01). Totally, the levels of SOD and GSH-Px in the periphery declined and MDA raised in the sham group and the model group, and the trend of this change was much more obvious in the model group.

### 3.3. Quantification of Neurotransmitters

Different standard solutions (containing IS) were diluted with 50% methanol (containing 0.2% formic acid) to six different concentrations. The calibration curves (each neurotransmitter peak area/the internal standard peak area (Yi/Ys) was plotted against the concentration) of neurotransmitters were obtained using the least-squares linear regression fit (*y* = *ax* + *b*) and *a* weighting factor of 1/*x*^2^. All the calibration curves indicated good linearity with correlation coefficients (*r*) ranging from 0.991 to 0.999. The limits of detection (LOD : S/N = 3) and the limits of quantification (LOQ : S/N = 10) were from 0.05 to 40.56 ng/mL and from 0.1 to 101.4 ng/mL, respectively. The precision of the method was determined using quality control (QC) samples (*n* = 6), and the results are summarized in Table 3. All the analytes showed relative standard deviation (RSD) below 15%.

**Figure 4:**
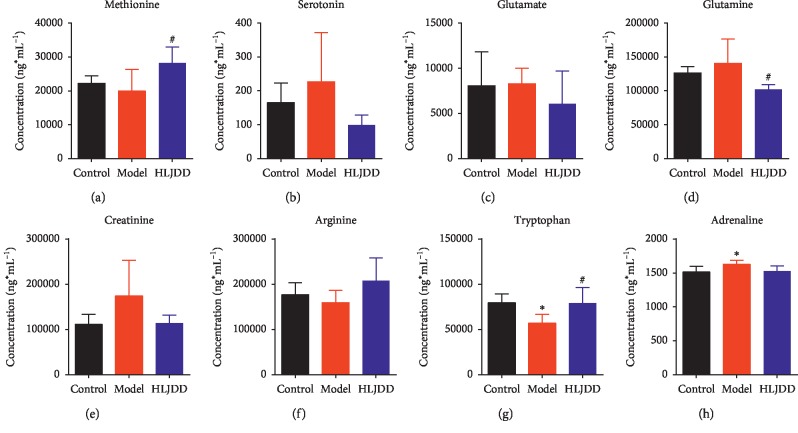
Changes of neurotransmitters in plasma of AD rats after HLJDD administration (*n* = 5). ^*∗*^*p* < 0.05 (comparison with control group); ^#^*p* < 0.05 (comparison with model group).

The contents of 8 neurotransmitters in the plasma were determined using UPLC-QqQ MS/MS. The contents of creatine, serotonin, adrenaline, glutamine, and glutamate displayed the same trend variation: increased in the model group while decreased in the HLJDD one-week group. Inversely, the contents of tryptophan, methionine, and arginine declined in the model group and raised in the HLJDD one-week group, particularly for tryptophan (Figure 4). Among them, the levels of tryptophan, methionine, and glutamine were reserved significantly after the administration of HLJDD.

### 3.4. Determination of AMPK and SIRT1 by Western Blot and Real-Time PCR

**Figure 5:**
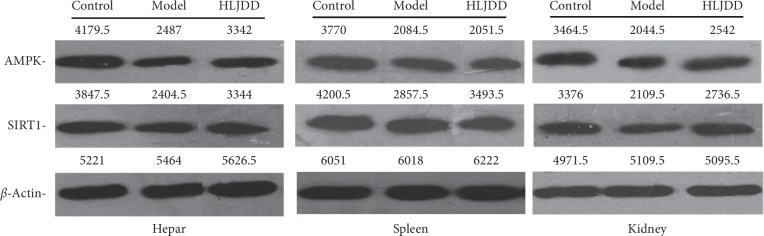
The expression of AMPK and SIRT1 in rats' hepar, spleen, and kidney with Western Blot analysis.

**Figure 6:**
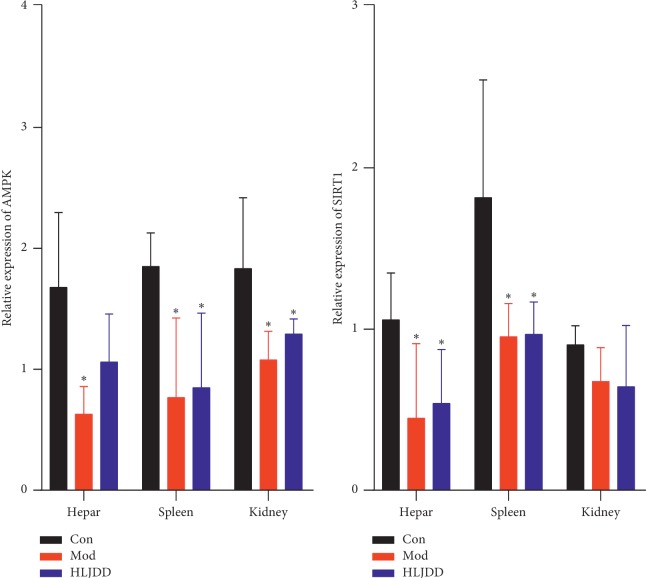
The expression of AMPK and SIRT1 in rat's hepar, spleen, and kidney with real-time *PCR* analysis (*n* = 5). Results were expressed as mean ± SD. ^*∗*^*p* < 0.05 (comparison with control group).

The levels of AMPK and SIRT1 in AD rats' hepar, spleen, and kidney were determined from the perspectives of protein and gene. According to the result of Western Blot (Figure 5), the protein levels of AMPK in hepar, spleen, and kidney declined in the model group and raised after HLJDD treatment. Interestingly, the protein expression trend of SIRT1 was the same as that of AMPK between these groups. As far as the result of the real-time *PCR* concerned (Table 4, Figure 6), the mRNA level of AMPK in rats' hepar, spleen, and kidney went down significantly (p < 0.05) in the model group and went up after the administration of HLJDD. Regarding the mRNA level of SIRT1, it drooped obviously after modelling and showed slight fluctuation in the HLJDD group.

## 4. Discussion

HLJDD, a traditional Chinese medicine, has widely been applied to treat cerebrovascular disease including dementia and ischemic stroke. Studies have demonstrated the great therapeutic effects of HLJDD in various AD models, including triple transgenic mice (3 × Tg-AD) and Tg-APP/PS1 mice [28, 29]. In this study, the rats injected with D-galactose and A*β*_25-35_-ibotenic acid were used as AD models. D-galactose, an aging accelerator, induces glycation end-products and causes oxidative stress [30] and was widely used in the construction of AD model [31, 32]. Intracerebral injections of A*β* and ibotenic acid in mice represent an acute and practical method to mimic AD pathology [33]. Plenty of evidences show that, in AD, a key factor in the accumulation of A*β* throughout the brain is the failure of neuroprotective microglia to remove extracellular amyloid [34, 35]. Overactive microglia and astrocytes clustered around A*β* plaques and secreted proinflammatory mediators [36, 37]. Compared with the control group and the sham group, rats in the model group showed worse memory impairment in the MWM test, which means that the model was successful.

In our previous study, 69 compounds of HLJDD were identified, mainly including iridoids, alkaloids, and flavonoids, and berberine is a representative element [3]. The achievements of berberine in the treatment of AD have been widely recognized [38, 39]. Due to the blood concentration of berberine, geniposide, and magnolia in AD rat induced by D-galactose and A*β*_25-35_-ibotenic acid tended to be constant in 5^th^ day [40], rats were sacrificed in the 7^th^ day. After administration for one week, there was a slight fluctuation in peripheric oxidative stress of the sham group compared to the control group, but it is not obvious. However, in the peripheral system, including plasma, hepar, spleen, and kidney, significantly decreased levels of SOD and GSH-Px and an increased contents of MDA were found in the model group by comparison with the control group. Combining with the MWM test, injection with D-galactose and A*β*_25-35_-ibotenic acid leads to oxidative stress disorder and cognitive impairment. HLJDD significantly lowered the levels of oxidative stress markers (MDA) in the peripheral system and enhanced the activities of antioxidases (SOD and GSH-Px), as compared with the model group. The cross talk between oxidative stress and A*β* deposition may occur via multiple ways affecting transcription of the APP gene or translation of APP mRNA [41]. Studies showed that the presence of methionine at 35 position of A*β* is critical to A*β*-induced oxidative stress and neurotoxicity [14, 42]. Severe methionine deficiency might cause dementia [43]. In this study, higher contents of methionine were found in the HLJDD group. Thus, alleviating oxidative stress and further reducing A*β* deposition may be the potential mechanism of HLJDD in treating AD.

Tryptophan, an essential amino acid, is the sole precursor of peripherally and produced serotonin (5-HT), and tryptophan metabolism by the Kynurenine (Kny) pathway generates neurotoxic metabolites [44]. Tryptophan depletion inhibits the levels of 5-HT and tryptophan in brain and reduced 5-HT_1A_ [45–47], which was responsible for cognitive impairment. In our study, HLJDD could reverse the decreased level of tryptophan in the AD model, which was possibly related to improving 5-HT synthesis and Kyn pathway metabolisms. The Kyn pathway may also be associated with inflammatory processes as well as the excitotoxic effects of glutamate [48]. In the present study, lower levels of glutamine and glutamate were found in the HLJDD group. Glutamine, a nonessential amino acid, becomes a conditionally essential amino acid in catabolic states due to the body's inability to synthesize sufficient amounts of glutamine during stress. The synthesis of glutamine is higher in rats of advanced age than that in youth, which may be considered an indicator of stress and frailty and, thus, the need for more glutamine [49]. Meanwhile, the glutaminase catalyzes the hydrolysis of glutamine to glutamate, while glutamine synthetase catalyzes the synthesis of glutamine from glutamate and ammonia. Together, the underling mechanism may be that the intervention of glutamine and tryptophan metabolism by HLJDD to reduce glutamate excitotoxicity.

In addition, AMPK-SIRT1 pathway may be closely related to oxidative stress, inflammation, and energy metabolisms. The increase in the expression of AMPK and SIRT1 by HLJDD may be owned to the reduction in oxidative stress observed in this model, which may furtherly influence the inflammation, and energy metabolism. AMPK, a major cellular energy sensor, plays a key role in cellular energy homeostasis. AMPK could regulate the expression of *α* and *β*-secretases, thus, affecting APP processing and A*β* generation [50]. SIRT1, an NAD^+^-dependent histone deacetylase, became famous molecules for slowing aging and decreasing age-related disorders. During oxidative stress, the NAD^+^-dependent DNA repair enzyme, poly (ADP-ribose) polymerase-1 (PARP), is activated and decreases NAD^+^ level which increases aging [51]. Decline in the SIRT1 activity in mice could be related to oxidative damage [52]. AMPK could activate SIRT1 by increasing NAD^+^ and NAD^+^/NADH and inverse SIRT1 by activating liver kinase B1 (LKB1) to react to AMPK [53]. It was reported that the AMPK-SIRT1 pathway had a significant regulatory effect on inflammation [54]. Study reported that HLJDD could inhibit the growth of hepatocellular carcinoma by activating AMPK-eEF2K [55]. Berberine, the main component in HLJDD, reduces A*β* deposition and decreases the expression of *β*-secretases via activating AMPK in neuroblastoma cells and primary cultured cortical neurons [38]. And even, baicalin is also reported as AMPK activators. Thus, regulating peripheric AMPK-SIRT1 pathway was probably one of the mechanisms of HLJDD in the treatment of AD rats, which involved the alleviation of oxidative stress, inflammation, and energy metabolism.

## 5. Conclusion

In this investigation, a method for the simultaneous measurement of 8 neurotransmitters in AD rat's plasma was established using an advanced technique with UPLC-QqQ MS/MS. Western Blot and Real-time PCR were used for the analysis of AMPK and SIRT1 to illuminate the mechanism underlying the anti-inflammation and regulating energy metabolism effects. The underlying therapeutic mechanism of HLJDD for the treatment of AD was associated with alleviating oxidation stress, inflammation, neurotransmitters, and energy metabolism. These data provide solid foundation for the potential use of HLJDD to treat AD.

## Figures and Tables

**Table 1:** The selected detecting ions, collision energy (CE), and scan width of the analytes.

	RT (min)	Chemical compounds	Quant/qual pair	Quant/qual CE(V)	Internal standard (IS)	Ion mode
IS 1	1.0	Diazepam	285 ⟶ 193/285 ⟶ 154	37/30	—	+
1	4.98	Creatinine	114 ⟶ 44/114 ⟶ 86	18/9	**IS1**	+
2	6.60	Serotonin	160 ⟶ 115/160 ⟶ 132	27/18	**IS 1**	+
3	8.08	Adrenaline	166 ⟶ 107/166 ⟶ 57	21/21	**IS 1**	+
4	9.00	Tryptophan	205 ⟶ 188/205 ⟶ 146	10/15	**IS 1**	+
5	10.08	Methionine	150 ⟶ 104/150 ⟶ 56	12/9	**IS 1**	+
6	14.94	Glutamine	147 ⟶ 84/147 ⟶ 130	9/18	**IS 1**	+
7	16.48	Glutamate	148 ⟶ 84/148 ⟶ 130	15/5	**IS 1**	+
8	19.18	Arginine	175 ⟶ 70/175 ⟶ 116	18/15	**IS 1**	+

Quant: quantitative; qual: qualitative.

**Table 2:** Primer sequences and real-time *PCR* conditions.

Gene	Primer sequences (5′ to 3′)	Base number	Product size	T annealing (°C)	Cycle
*β*-Actin	F: GAAGTGTGACGTTGACATCCG	21	282 bp	60	40
	R: GCCTAGAAGCATTTGCGGTG	20		60	40

AMPK	F: TCTCGGGGTGGTTCGGTG	18	178 bp	60	40
	R: GGGGACAGGATTTTCGGATT	20		60	40

SIRT1	F: TTCACCACAAATACTGCCAAGA	22	218 bp	60	40
	R: GATACATTACACCAAATCCTCAACA	25		60	40

**Table 3:** The regression equations, LOD, LOQ, and linear range of the 8 analytes.

Compounds	Regression equations	*R* ^2^	LOD (ng/mL)	LOQ (ng/mL)	Linear range (ng/mL)	Repeatability (RSD, %)		
						Low concentration	Middle concentration	High concentration
Creatinine	*Y* = 0.013015 *∗* *X* + 0.355134	0.999	0.05	0.10	39.24–39240	4.54	3.57	5.43
Serotonin	*Y* = 0.050158 *∗* *X* − 6.815533 × 10^−4^	0.998	1.01	2.02	4.048–404.8	12.21	1.58	2.42
Adrenaline	*Y* = 0.004093 *∗* *X* + 3.553836 × 10^−4^	0.991	9.85	39.40	78.80–3940	4.16	3.27	2.31
Tryptophan	*Y* = 0.003530 *∗* *X* − 0.009977	0.993	0.30	1.02	421.2–25272	1.98	1.88	2.05
Methionine	*Y* = 2.488989 × 10^−4^ *∗* *X* + 6.677709 × 10^−4^	0.995	40.16	80.32	160.64–8032	7.02	1.28	2.53
Glutamine	*Y* = 7.373791 × 10^−4^ *∗* *X* − 7.910165 × 10^−4^	0.999	40.56	101.40	405.6–40560	13.52	10.62	8.91
Glutamate	*Y* = 0.003640 *∗* *X* + 0.003405	0.998	1.12	2.40	119.64–2991	8.12	5.43	3.13
Arginine	*Y* = 0.003604 *∗* *X* + 4.417252 × 10^−5^	0.992	0.15	0.50	203.8–48912	7.47	3.34	3.28

**Table 4:** The relative expression of AMPK and SIRT1 in rat tissues (*n* = 5).

Genes	Hepar			Spleen			Kidney		
	Control group	Model group	HLJDD group	Control group	Model group	HLJDD group	Control group	Model group	HLJDD group
AMPK	1.68 ± 0.62	0.63 ± 0.23^*∗*^	1.06 ± 0.40	2.54 ± 1.56	0.77 ± 0.66^*∗*^	0.85 ± 0.62^*∗*^	1.83 ± 0.58	1.08 ± 0.24^*∗*^	1.29 ± 0.12^*∗*^
SIRT1	1.06 ± 0.29	0.45 ± 0.46^*∗*^	0.54 ± 0.34^*∗*^	1.81 ± 0.73	0.95 ± 0.21^*∗*^	0.97 ± 0.20^*∗*^	0.90 ± 0.12	0.68 ± 0.21	0.64 ± 0.38

^*∗*^ *p* < 0.05 (comparison with control group).

## Data Availability

The data used to support the findings of this study are included within the article.

## Abbreviation

5-HT: Serotonin
AD: Alzheimer disease
AMPK: AMP-activated protein kinase
A*β*: Amyloid beta-peptide
BSA: Bovine serum albumin
GSH-Px: Glutathione peroxidase
HLJDD: Huang-Lian-Jie-Du Decoction
HLJDD-EP: HLJDD extract power
Kny: Kynurenine
LKB1: Liver kinase B1
MDA: Malondialdehyde
MRM: Multiple reaction monitory
NBM: The nucleus basalis magnocellularis
NFTs: Senile plaques and neurofibrillary tangles
QC: Quality control
SIRT1: Silent information regulator of transcription 1
SOD: Superoxide dismutase
TCM: Traditional Chinese medicine.
